# Meta-analysis of randomized clinical trials in the era of individual patient data sharing

**DOI:** 10.1007/s10147-018-1237-z

**Published:** 2018-01-12

**Authors:** Takuya Kawahara, Musashi Fukuda, Koji Oba, Junichi Sakamoto, Marc Buyse

**Affiliations:** 1 Department of Biostatistics, School of Public Health, Graduate School of Medicine, The University of Tokyo, Annex of Bldg. 3, 5th Floor, 7-3-1 Hongo, Bunkyo-ku, Tokyo, 113-0033 Japan; 2 Astellas Pharma Inc., Tokyo, 2-5-1 Nihonbashi-Honcho, Chuo-ku, Tokyo, 103-8411 Japan; 3 Tokai Central Hospital, 4-6-2 Sohara Higashijima-cho, Kakamigahara, Gifu, 504-8601 Japan; 4 International Drug Development Institute (IDDI) Inc., San Francisco, CA 94109 USA

**Keywords:** Data sharing, Individual patient data, Meta-analysis

## Abstract

**Background:**

Individual patient data (IPD) meta-analysis is considered to be a gold standard when the results of several randomized trials are combined. Recent initiatives on sharing IPD from clinical trials offer unprecedented opportunities for using such data in IPD meta-analyses.

**Methods:**

First, we discuss the evidence generated and the benefits obtained by a long-established prospective IPD meta-analysis in early breast cancer. Next, we discuss a data-sharing system that has been adopted by several pharmaceutical sponsors. We review a number of retrospective IPD meta-analyses that have already been proposed using this data-sharing system. Finally, we discuss the role of data sharing in IPD meta-analysis in the future.

**Results:**

Treatment effects can be more reliably estimated in both types of IPD meta-analyses than with summary statistics extracted from published papers. Specifically, with rich covariate information available on each patient, prognostic and predictive factors can be identified or confirmed. Also, when several endpoints are available, surrogate endpoints can be assessed statistically.

**Conclusions:**

Although there are difficulties in conducting, analyzing, and interpreting retrospective IPD meta-analysis utilizing the currently available data-sharing systems, data sharing will play an important role in IPD meta-analysis in the future.

## Introduction

In 2009, a special issue of the *International Journal of Clinical Oncology* reviewed the implementation and limitations of the meta-analysis of randomized controlled trials (RCTs). This special issue included a general discussion of the role of meta-analysis [1], implementation of the tabulated-data meta-analysis [2], additional contributions of individual patient data (IPD) meta-analysis [3], and the development of the statistical methods for performing IPD meta-analysis [4]. In these articles the benefits of IPD meta-analysis were discussed, and IPD meta-analysis was presented as a gold standard for conducting a quantitative review of evidence arising from randomized clinical trials.

Historically, meta-analysis started in the early 1980s when it became apparent that many randomized trials were too small to reliably establish the effects, or lack thereof, of most treatments of patients with cancer and cardiovascular diseases [5, 6]. The need to combine data from several trials led to early meta-analyses based on summary statistics extracted from the published results of randomized trials. Such meta-analyses suffered from poorly standardized data, publication bias, selective reporting, and other sources of bias, such as post hoc choices of the hypotheses tested. The pioneering work of the Early Breast Cancer Trialists’ Collaborative Group (EBCTCG) led to the creation of several research groups with the aim to conduct IPD meta-analyses. These groups started with retrospective meta-analyses and then continued with prospective meta-analyses of trials conducted by the same investigators or research groups. A prospective meta-analysis is a meta-analysis in which eligible randomized trials are identified before their results are known. In comparison, in a retrospective meta-analysis efforts are made to identify all ongoing trials, both to maximize precision and to avoid publication bias [7]. A prospective meta-analysis may be limited to a small number of investigators or research groups who standardize outcomes and case report forms used to collect the data.

More recently, the concept of sharing IPD from clinical trials has attracted much attention in major journals as well as in some regulatory agencies [8–17]. The current trend toward sharing data has the potential to revolutionize IPD meta-analysis if the data-sharing policy is successfully implemented in the years to come. Our purpose in this paper is to discuss the respective advantages and limitations of retrospective IPD meta-analyses utilizing data-sharing systems as compared with those of prospective meta-analyses conducted by large collaborative groups. We first introduce the EBCTCG as an example of prospective IPD meta-analysis to focus on the evidence this group has generated over the last three decades. Next, we review the data-sharing system and discuss examples of a retrospective IPD meta-analysis that uses this data-sharing system. Finally, we discuss the role of data sharing for IPD meta-analysis in the future.

## Example of prospective IPD meta-analysis: EBCTCG

The EBCTCG is one of the world’s largest meta-analysis groups (Table 1). It was initiated in 1985 with the aim of collaborating the work of various research groups studying the treatment of early breast cancer and currently involves the collaboration of hundreds of such research groups from around the world [20]. Over the last 30+ years, the EBCTCG has identified more than 600 randomized trials of treatments for women with operable breast cancer and collected IPD from more than 400 of these, involving a total of 460,000 women. In the early 1990s, the EBCTCG routinely sought IPD from every randomized trial that had compared treatments for women with operable breast cancer in which recurrence or mortality was a principal outcome. The comparisons being tackled in the present decade relate to hormonal therapy, chemotherapy, other systemic therapies, and local therapy, in which death from secondary cancers, non-breast-cancers, and/or cardiovascular diseases are principal outcomes in addition to recurrence or mortality. The study results of the EBCTCG can be obtained at the webpage of the Clinical Trial Service Unit and Epidemiological Studies Unit [21].

**Table 1:** Examples of prospective individual patient data meta-analysis groups

Group	Patient population	Main outcome	Formal protocol
Early Breast Cancer Trialists’ Collaborative Group (EBCTCG)	Breast cancer	Overall survival	On request
Blood Pressure Lowering Treatment Trialists’ Collaboration (BPLTTC)	Hypertension	Major cardiovascular events	Yes [18]
Chemoradiotherapy for Cervical Cancer Meta-Analysis Collaboration	Cervical cancer	Overall survival	On request
NSCLC Meta-analyses Collaborative Group	Non-small-cell lung cancer	Overall survival	On request
Cholesterol Treatment Trialists’ (CTT) Collaborators	Hyperlipidemia	Major vascular events	Yes [19]

The variables that are to be provided by the collaborators are specified on this webpage, namely, patient characteristics, surgical details, nodal status, tumor characteristics, receptor status, non-compliance before any recurrence, cancer recurrence and second cancers, survival, additional tumor marker data, bone fractures and cardiovascular events, and trials with neo-adjuvant systemic therapy. Each collaborator prepares the data formatted as “one record for each woman,” and the statistical group in Oxford conducts the meta-analysis in cooperation with the steering committee of EBCTCG. What kind of evidence can be obtained through the above prospective collaboration and preparation of the standardized data as indicated? As an example of prospective IPD meta-analysis, we introduce one of the results of the EBCTCG [22], which highlights a number of important components of prospective IPD meta-analysis.

The study was a meta-analysis of all RCTs which targeted effective adjuvant therapy for early breast cancers and had been initiated from 1973 to 2003. Two primary endpoints were death from breast cancer and death from any cause. The meta-analysis included data from as many as 100,000 patients collected between 2005 and 2010 and could not have been done without an IPD meta-analysis; in other words, such an analysis based on latest data cannot be done with a tabulated-data meta-analysis. Moreover, the study collaborators were requested to send the IPDs of “ever randomized patients,” which included any woman who was randomized and then was later categorized as ineligible, withdrawn, unevaluable, lost to follow-up, or protocol deviant. Having data on all “ever randomized patients,” investigators can assess the impact of excluding some patients from the meta-analysis (selection bias), which is only possible when IPD are available.

While the main object of the IPD meta-analysis was to compare the treatment regimens for all early breast cancer patients included in the individual studies, the IPD also included covariates which affect survival; hence, the meta-analysis could also assess treatment effects in various patient subgroups. The study reported that the treatment regimens scarcely affected the overall survival between the subgroups of age, status of lymph nodes, tumor size, differentiation, estrogen receptor, or tamoxifen history, based on the Chi-squared statistic of heterogeneity (Fig. 6 of [22]).

Anthracycline-based regimens are known to have adverse cardiac effects, but the incidence was too small to be estimated reliably in individual trials. In the IPD meta-analysis, untoward effects of anthracycline-based regimens could be estimated accurately ([22], pp. 441–442).

This example of a prospective IPD meta-analysis performed by the EBCTCG illustrates many important advantages of IPD meta-analysis, in particular the absence of publication bias because of the prospective nature of the meta-analysis. However, a long and difficult process that can take several years and require a sophisticated organization is one of the limitations of prospectively obtaining IPD. Authors have been conducting retrospective IPD meta-analysis for over 15 years but it is also difficult and time-consuming to obtain IPD for a retrospective IPD meta-analysis [23–27]. Recently, however, data-sharing initiatives have been discussed in the medical literature [8–17]. If data sharing can be put into practice, retrospective IPD meta-analysis may be the more efficient option compared to (or sometimes in addition to) prospective ones in terms of conducting an IPD meta-analysis.

## Data sharing and retrospective IPD meta-analysis

Sharing IPD from clinical trials is an increasing trend among stakeholders involved in medical research [28–34]. The European Medicines Agency (EMA) published a draft “Policy on publication and access to clinical-trial data: Policy 0070” in June 2013, with finalization of the policy in October 2014 [35]. In January 2014, the European Federation of Pharmaceutical Industries and Association (EFPIA) and the Pharmaceutical Research and Manufacturers of America (PhRMA) jointly implemented “Principles for Responsible Clinical Trial Data Sharing” [36]. In these Principles, enhancing the sharing of anonymized patient-level and study-level data with qualified researchers is described as one of the commitment of biopharmaceutical companies. In January 2016, the International Committee of Medical Journal Editors (ICMJE) proposed that sharing of the de-identified IPD of any clinical trial submitted for publication to an ICMJE journal no later than 6 months after publication should be a prerequisite for publication [14]. More recently, the ICMJE decided that after 1 July 2018 all authors submitting a clinical trial study for publication have to agree to these data-sharing statements at the time of manuscript submission [15]. In line with these policies, most pharmaceutical companies have launched data-sharing initiatives. Some companies share the data on their own webpages, while others utilize a common system for data sharing, such as Clinical Study Data Request (CSDR) [37], Yale University Open Data Access (YODA) [38] and Project Data Sphere [39].

CSDR is one of the largest common multi-sponsor systems for data sharing, being initiated by GlaxoSmithKline (GSK) [40]. Thirteen pharmaceutical companies have participated in this system up to August 2017, and overviews of their activity during the first 2 years have been published [41, 42]. Researchers can use the CSDR webpage to request access to anonymized IPD and supporting documents from clinical studies conducted by study sponsors that are committed to participate in CSDR. In order to request IPD from the studies listed on the website, researchers have to submit their research proposals, including a statistical analysis plan. The request for IPD after submission of research proposals is assessed as follows: (1) requirement checks; (2) review by independent review panel; (3) data sharing agreement; (4) de-identified data preparation by the sponsor; and (5) data analysis. The details of a research proposal which has been approved for data sharing can be followed on the webpage (see Metrics in [37]). The types of research projects are very diverse, including re-analysis of the data of the published study and meta-analysis based on either tabulated data or IPD. Of the 174 research proposals approved of up to 31 August 2017, 12 proposals were IPD meta-analysis, including network meta-analysis (Table 2).

**Table 2:** List of approved research proposals for meta-analysis in the Clinical Study Data Request

Title	Lead researcher	Date(s)	Sponsors^a^	*N*	Type^b^
Use of Hhparins in patients with cancer: individual patient-data meta-analysis of randomized trials	Holger Schunemann	July 2014–March 2016	GSK, Sanofi	2	IPD
Nocebo effects in the treatment of bipolar disorder: results from an individual study participant level meta-analysis of the placebo arm of olanzapine clinical trials	Seetal Dodd	October 2014	Lilly	9	IPD
Ritonavir-boosted protease inhibitor-based versus non-nucleoside reverse transcriptase inhibitor-based highly active antiretroviral therapy regimens: a systematic review and individual patient data meta-analysis	Alvaro H Borges	August 2014	ViiV	1	IPD
Initial severity and antidepressant efficacy for anxiety disorders: an individual patient data meta-analysis	Ymkje Anna de Vries	June 2015	GSK/Lilly	29	IPD
Incidence of brain metastases in metastatic breast cancer patients receiving palliative chemotherapy with or without bevacizumab: a meta analysis of five prospective randomized phase III trials	Rupert Bartsch	March 2016	Roche	5	SUM
An independent evaluation of the cardiovascular risk of rosiglitazone: meta-analysis of GSK’s publicly available clinical trial data	Joseph S. Ross	September 2015	GSK	59	SUM
A meta-analysis of Parkinson’s disease	Atul Butte	August 2015	BI/GSK/UCB	25	SUM
Initial severity and efficacy of antipsychotics for schizophrenia and bipolar mania: individual participant level analyses of placebo-controlled studies	Stefan Leucht	February 2015	Lilly	7	SUM
Correlation between SF-36 PCS and MCS scores and SELENA SLEDAI activity score in SLE: a meta-analysis	Chaigne Benjamin	–	GSK	3	SUM
Assessing the comparative efficacy of therapeutics for chronic obstructive pulmonary disease (COPD): a meta-analysis of randomized trials	Kristen M. Sweet	June 2015	BI/GSK	25	SUM
Meta-analysis of the risk of relapse in bipolar disorder	Joaquim Radua	January 2015	Lilly	1	IPD
Sex-specific efficacy of mirabegron for overactive bladder (OAB) symptoms: individual patient data meta-analyses	Marco H. Blanker	June 2017	Astellas	10	IPD
A meta-analysis of tardive dyskinesia rates in studies comparing second generation antipsychotics (SGAs) with each other or to first-generation antipsychotics (FGAs)	Christoph U Correll	October 2016	Lilly	8	SUM
ANSELMA: Antiangiogenic agents in advanced non-small cell lung cancer patients who failed first-line chemotherapy: an individual patient data meta-analysis	Benjamin Besse	April–August 2017	Roche Sanofi	2 3	IPD
Individual participant data meta-analysis of antidepressant trials for major depression In Japan	Shigeto Yamawaki	May 2017	GSK	2	IPD
Multivariate methods for meta-analysis of vaccine studies with multiple correlated outcomes	Merryn Voysey	July 2015	GSK	49	IPD
Antiepileptic drug monotherapy for epilepsy: an overview of systematic reviews and network meta-analysis	Antony Marson	May 2014	GSK	1	IPD-NMA
PhD project: Bayesian multivariate network meta-analysis of ordered categorical data	Fabrizio Messina	October 2014	Roche	2	IPD-NMA
A systematic literature review and Individual patient-level data enhanced network meta-analysis to determine the comparative efficacy and safety of first-line HIV antiretroviral therapies	Steve Kanters	May 2017	ViiV	3	IPD-NMA

*Date* Date of data sharing agreement, *N* number of studies

^a^*GSK* GlaxoSmithKline,* ViiV* ViiV Healthcare,* UCB* Union Chimique Belge,* BI* Boehringer Ingelheim GmbH

^b^*IPD* individual patient data (meta-analysis based on IPD), *SUM* summary statistics (meta-analysis based on SUM), *IPD-NMA* network meta-analysis based on IPD

Using the IPD of previous studies through a system of data sharing, such as CSDR, researchers can conduct retrospective IPD meta-analyses. In contrast to a prospective IPD meta-analysis, such as that conducted by EBCTCG, each study shared in the system is not intended to be used in the meta-analysis and, therefore, variables included in each study are not necessarily consistent across different studies.

An example of a retrospective IPD meta-analysis currently in progress is that of Schünemann et al. who initially published the design of their research project using data obtained through CSDR [43]. Subsequent to their previous study-level systematic review and meta-analysis to evaluate the role of parenteral anticoagulants in patients with cancer, the results of which suggest a survival benefit and a reduction in venous thromboembolism, these researchers are conducting an IPD meta-analysis to further investigate the questions that remain unanswered. Specifically, their primary objective is to explore the magnitude of the survival benefit of parenteral anticoagulants and to address which subgroups of patients with cancer are more likely to benefit from parenteral anticoagulants. To this end, they identified 15 randomized trials that fulfill their eligibility criteria. Two of these are among the studies available on CSDR. Consequently, these investigators submitted research proposals and requested anonymized data from the studies. Their requests were agreed to in July 2014 for the INPACT Study [44] (sponsored by GSK) and in March 2016 for the SAVE-ONCO Study [45] (sponsored by Sanofi). For the other studies deemed eligible for inclusion in their meta-analysis (13 of 15 trials), Schünemann and colleagues contacted the authors of each study directly and requested them to share their data. Using the shared IPD, their research project is currently in progress. For the analysis of mortality, the primary outcome, multilevel models will be used that include patient-level variables, such as comorbidities and age, as fixed effects and trial as a random effect. In order to test for a differential treatment effect among the various pre-specified subgroups defined by type and stage of cancer or concomitant treatment, tests of interaction will be conducted. In addition, a risk-prediction model for venous thromboembolism in patients with cancer will be developed using logistic regression or Cox proportional hazards regression. With data from a total of more than 9000 participants, the strength of their IPD meta-analysis is the possibility of identifying patient-level effect modifiers and exploring more precisely the survival benefit suggested by their previously published tabulated-data meta-analysis.

## Discussion

We have reviewed IPD meta-analyses conducted prospectively and retrospectively. The characteristics of prospective and retrospective IPD meta-analysis compared to tabulated-data meta-analysis are summarized in Table 3. In both types of IPD meta-analyses, the availability of detailed covariate information for each patient allows investigators to examine the effect of risk factors and treatment effect modifiers.

**Table 3:** Characteristics of different types of meta-analyses

Type of meta-analysis	Conduct	Analysis	Interpretation
Prospective meta-analysis using IPD	Adds little cost and complexity to the conduct of individual trials	Enhances power and adds pre-specified analyses to individual trials	Enriches interpretation of individual trials
Retrospective meta-analysis using IPD	Difficult and time-consuming to obtain IPD	Enhances power and adds post hoc analyses to individual trials	Enriches interpretation of individual trials
Retrospective analysis using summary data	Easy access to summary statistics on primary outcome, but not on subsets or secondary outcomes	Enhances power and allows exploration of heterogeneity between trials, but otherwise limited	Very limited and often unreliable

In order to conduct IPD meta-analysis, it is often necessary to put in place a large collaborative group. Large and long-lasting collaborative groups such as the EBCTCG have enabled investigators to examine important research questions that could not be answered in individual studies. However, such a prospective IPD meta-analysis also takes much time and adequate financial resources, not only to fund the central office of the IPD meta-analysis, but also to fund all the groups who share their data.

To the contrary, it is reasonable to expect that a retrospective IPD meta-analysis will be easy to conduct in the era of data sharing. Nevitt et al. recently conducted a systematic review of 760 IPD meta-analyses and only 25% of these retrieved 100% of eligible IPD for the respective analysis [46]. With a system of data sharing such as CSDR, it may be possible for researchers to use IPD of previous studies without the overhead of a large collaborative group. Very few studies of IPD meta-analysis using CSDR have been reported to date [47–49]. In the case of CSDR, researchers can submit requests to sponsors to inquiry about the availability of data from studies that are not listed on the website; however, the number of such inquiries has thus far been surprisingly small (See Metrics on CSDR webpage). It is likely that a retrospective IPD meta-analysis using a data sharing system is more vulnerable to publication bias, if investigators use a sample of convenience (studies available on a data sharing system) rather than the totality of all trials ever conducted on the question of interest.

Despite its scientific merits of data sharing, it is always associated with the protection of sensitive personal information. The draft policy of the EMA acknowledges the importance of personal data protection when sharing IPD [35], but the procedures by which an acceptable level of protection can be achieved are as yet unclear. In Japan, the ethical guidelines for medical and health research involving human subjects issued by the Ministry of Education, Culture, Sports, Science and Technology/Ministry of Health, Labour and Welfare in 2015 contained no official statement of data sharing [50]; however, amendments in 2017 included the amendment of the act on the Protection of Personal Information. A well-balanced discussion is expected, and such a discussion may be imperative for encouraging data sharing and reaching a balance between the protection of personal data and scientific research value.

Data sharing has just started, and very few IPD meta-analyses using shared data have been conducted. Data sharing will accelerate access to the IPD of clinical trials, and a corresponding increase in the number of IPD meta-analyses conducted in the future can be expected. In conclusion, although there are some difficulties in conducting, analyzing, and interpreting retrospective IPD meta-analysis utilizing data sharing systems, data sharing most certainly will play an important role in IPD meta-analysis.
